# Commuters’ Exposure to Particulate Matter Air Pollution Is Affected by Mode of Transport, Fuel Type, and Route

**DOI:** 10.1289/ehp.0901622

**Published:** 2010-02-25

**Authors:** Moniek Zuurbier, Gerard Hoek, Marieke Oldenwening, Virissa Lenters, Kees Meliefste, Peter van den Hazel, Bert Brunekreef

**Affiliations:** 1 Public Health Services Gelderland Midden, Arnhem, the Netherlands; 2 Institute for Risk Assessment Sciences, Division of Environmental Epidemiology, Utrecht University, Utrecht, the Netherlands and; 3 Julius Center for Health Sciences and Primary Care, Utrecht University, Utrecht, the Netherlands

**Keywords:** air pollution, commuting, cycling, particles, soot, transport

## Abstract

**Background:**

Commuters are exposed to high concentrations of air pollutants, but little quantitative information is currently available on differences in exposure between different modes of transport, routes, and fuel types.

**Objectives:**

The aim of our study was to assess differences in commuters’ exposure to traffic-related air pollution related to transport mode, route, and fuel type.

**Methods:**

We measured particle number counts (PNCs) and concentrations of PM_2.5_ (particulate matter ≤ 2.5 μm in aerodynamic diameter), PM_10_, and soot between June 2007 and June 2008 on 47 weekdays, from 0800 to 1000 hours, in diesel and electric buses, gasoline- and diesel-fueled cars, and along two bicycle routes with different traffic intensities in Arnhem, the Netherlands. In addition, each-day measurements were taken at an urban background location.

**Results:**

We found that median PNC exposures were highest in diesel buses (38,500 particles/cm^3^) and for cyclists along the high-traffic intensity route (46,600 particles/cm^3^) and lowest in electric buses (29,200 particles/cm^3^). Median PM_10_ exposure was highest from diesel buses (47 μg/m^3^) and lowest along the high- and low-traffic bicycle routes (39 and 37 μg/m^3^). The median soot exposure was highest in gasoline-fueled cars (9.0 × 10^−5^/m), diesel cars (7.9 × 10^−5^/m), and diesel buses (7.4 × 10^−5^/m) and lowest along the low-traffic bicycle route (4.9 × 10^−5^/m). Because the minute ventilation (volume of air per minute) of cyclists, which we estimated from measured heart rates, was twice the minute ventilation of car and bus passengers, we calculated that the inhaled air pollution doses were highest for cyclists. With the exception of PM_10_, we found that inhaled air pollution doses were lowest for electric bus passengers.

**Conclusions:**

Commuters’ rush hour exposures were significantly influenced by mode of transport, route, and fuel type.

In Europe, people spend 1–1.5 hr/day traveling (World Health Organization 2005). Because most of the traveling occurs during rush hours, commuting contributes significantly to total exposure to transport-related air pollution. Exposure to air pollution in traffic has been related to short-term cardiovascular and respiratory health effects in a small number of studies (Adar et al. 2007; McCreanor et al. 2007; Peters et al. 2004; Riediker et al. 2004; Strak et al. 2009).

Air pollution levels in various transport modes have been compared in a limited number of studies. In most studies, exposure to particulate matter was higher in buses and cars than were exposures encountered during walking or cycling (Kaur et al. 2007). Rank et al. (2001) measured higher total dust concentrations when driving in cars than when riding on bicycles. Adams et al. (2001a, 2002) detected highest exposures to PM ≤ 2.5 μm in aerodynamic diameter (PM_2.5_) in buses, followed by cars, and lowest on bicycles. They found that elemental carbon (EC) exposure was highest when riding in cars, followed by buses, and lowest when riding on bicycles. In another study, McNabola et al. (2008) found that PM_2.5_ exposures were highest in buses, followed by cars and bicycles, and lowest when walking. In contrast, Briggs et al. (2008) found exposures to coarse, fine, and ultrafine particles to be higher when walking on sidewalks than when driving in a car. Boogaard et al. (2009) found slightly higher particle number counts (PNCs) and PM_2.5_ exposure levels when driving in cars than when riding on bicycles.

Exposure levels are influenced by the mode of transport and by the route and type of vehicle. For walkers and cyclists, PM exposures are higher on high-traffic routes than on low-traffic routes (Adams et al. 2001a; Kaur et al. 2005; McNabola et al. 2008; Strak et al. 2009; Thai et al. 2008). Adams et al. (2001a) measured higher PM_2.5_ levels in open-back buses than in closed buses, and Hammond et al. (2006) measured three to four times higher PNC in diesel buses than in compressed natural gas buses or buses with oxidation catalysts. Several studies concluded that a significant portion (up to 30%) of air pollutants in (school) buses is due to self-pollution (Adar et al. 2008; Behrentz et al. 2004). For example, opening windows during driving, idling of the bus, and opening bus doors led to higher in-bus exposures (Asmi et al. 2009; Hammond et al. 2006).

It is currently difficult to precisely quantify differences in air pollution exposure between different modes of transport. One reason is that in most studies, exposures in different modes were not measured simultaneously; thus, background concentrations may have been different. Some studies reported air pollution levels from background monitoring stations to indicate different conditions between the different sampling days (Adams et al. 2001a; Fondelli et al. 2008; Thai et al. 2008). However, different particle samplers often were used to measure exposure of commuters and at fixed sites (typically routine monitors), which made quantitative comparisons between commuters’ exposure and urban background sites difficult.

To overcome some of the limitations of previous studies, we decided to conduct a larger scale study that systematically compares exposure levels between different modes of transport. The aim of the present study was to quantify differences in exposure to air pollutants in traffic compared with simultaneously measured urban background concentrations and to examine the differences in air pollution exposure associated with commuting by car, bus, and bicycle.

## Materials and Methods

The TRAVEL (Transport Related Air Pollution, Variance in Commuting, Exposure and Lung Function) study examined commuters’ exposure to air pollution and associated health effects. Exposures to PNC, PM_2.5_, PM_10_, and soot were measured in diesel and gasoline-fueled cars, in diesel and electric trolley buses, and along two bicycle routes with low- and high-traffic intensity. To determine doses of inhaled air pollution, we estimated minute ventilation (ventilation rate per minute) for the three commuting modes by measuring the heart rates of 34 volunteers. The relation between heart rate and minute ventilation was determined for each volunteer (Zuurbier et al. 2009).

### Transport modes and urban background

We collected particle samples between 0800 and 1000 hours on 47 weekdays (Tuesdays and Thursdays), with samples collected for each mode of transportation on approximately one-third of the days. Collection days were evenly spaced over 1 year, from June 2007 until June 2008, to include a range of meteorological conditions.

In total, we had 47 sampling days; of these, one-third of the collections were made in cars, one-third in buses, and one-third on bicycles. The measurements were taken in varying order. Thus, on one-third of the days, we measured exposures simultaneously in a diesel and a gasoline-fueled car. The cars were new rental, multiperson vehicles; we used two diesel models—the Ford S-Max and the Volkswagen Sharan, and four gasoline-fueled models—Ford Galaxy, Renault Espace, Seat Alhambra, and Peugeot 807. Car windows were closed during sampling, and air conditioning was set at a moderate level.

On one-third of the days we measured exposures in an electric trolley bus and a diesel bus simultaneously, during the regular service of the buses; hence, during the trip passengers got on and off. The buses were not equipped with air conditioning, windows were closed during sampling, and smoking was not allowed. The mean age of both diesel and electric buses was 7 years old (the two newest buses were 5 years old, the two oldest buses were 18 and 10 years old). Diesel buses were retrofitted with particulate filters.

On the remaining one-third of the days we measured exposures simultaneously along two cycling routes with different traffic intensity. Technicians rode three-wheeled cargo bicycles to transport the equipment.

**Figure 1:**
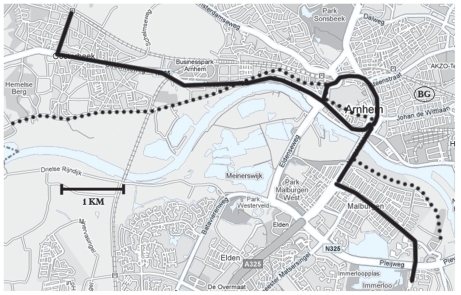
Map of routes. BG, urban background measurement site; dotted line, low-traffic bicycle route; solid line, car, bus, and high-traffic bicycle route. ©2009 Google – Map data ©2009 Tele Atlas.

The 22-km route was the same for the cars, buses, and the high-traffic bicycle route. It covered the center of Arnhem, a medium-sized Dutch city (145,000 inhabitants), and followed roads with an average traffic intensity of 15,000 vehicles/day (range, 7,000–30,000 vehicles/day). To examine the difference in exposure when cycling along high- and low-traffic roads, the second bicycle route followed roads with less traffic, at some distance from the other route (Figure 1). We selected the streets with the least amount of traffic to maximize the contrast with the high-traffic route. Average traffic intensity on this 22-km route was approximately 5,000 vehicles/day and varied from car-free roads to roads with 30,000 vehicles/day on small sections of the route that overlapped with the high-traffic route. The cars drove the route twice, the buses went around one and two-third times, and the bicycles were driven around once and doubled a small portion (10%) of the route to complete 2 hr.

On each sampling day, at the same time interval, we collected samples at an urban background location that was situated near the start and end point of the route, 250 m from main roads (Figure 1), using the same types of samplers.

### Measurement methods

We measured PNCs with three portable, real-time condensation particle counters (CPCs; model 3007, TSI, Inc., Shoreview, MN, USA). This CPC uses 1-propanol as condensation liquid and measures particles > 10 nm. One-second averages were recorded. We compared quality assurance tests on the three CPC3007 units with one TSI CPC3022 unit [see Supplemental Material (doi:10.1289/ehp.0901622)].

We measured PM_2.5_ using real-time active-sampling personal DataRAMs (model 1200; MIE Inc., Bedford, MA, USA), equipped with PM_2.5_ cyclones (model GK 2.05 KTL; BGI Inc., Waltham, MA, USA) and pumps with a flow of 4 ± 0.4 L/min (AFC400s, BGI Inc.). The DataRAMs were calibrated by the manufacturer against SAE fine (ISO fine) (International Organization for Standardization, Geneva, Switzerland) dust. One-second averages were recorded. Before each measurement, we performed a zero check using a high-efficiency particulate (HEPA) filter (provided by TSI). A correction factor was used to correct for relative humidity (RH) [see Supplemental Material (doi:10.1289/ehp.0901622)].

We collected PM_10_ on 37-mm 2-μm pore-size Teflon filters, using Harvard Impactors (Air Diagnostics and Engineering Inc., Naples, ME, USA) and pumps (model SP-280E, Air Diagnostics and Engineering Inc., Harrison, ME ) with a flow of 10 ± 0.5 L/min.

We determined soot content of the filters using a smoke stain reflectometer (model M43D, Diffusion Systems Ltd., London, UK). We measured reflectance at five different positions on each filter and calculated the average into an absorbance coefficient according to ISO 9835 (Cyrys et al. 2003). To facilitate calculation of doses, we converted soot (absorption) levels into EC concentrations using the equation EC = 1.6053 × absorption – 0.2620 (Cyrys et al. 2003). Exposures are expressed as soot (absorption) levels and doses as EC.

Additional details on quality assurance are reported in the Supplemental Material (doi:10.1289/ehp.0901622).

We used voice recorders on 3 of the 16 bicycle sampling days to record events such as passing mopeds and passing buses. We obtained meteorological data (temperature, rain, wind speed, wind direction) from a meteorological station near the city center of Arnhem.

To estimate the minute ventilation during commuting by bicycle, car, and bus, heart rates of the participants were recorded during commuting using Polar RS400 heart rate monitors (Polar Electro, Kempele, Finland). All participants performed a submaximal bicycle ergometer test during which heart rate and minute ventilation were measured simultaneously at increasing cycling intensity. Minute ventilation was measured using a pneumotachometer (Jaeger, Viasys Healthcare, Hoechberg, Germany). The tests have been described in more detail elsewhere (Zuurbier et al. 2009). We used the mean ventilation rates of the participants to calculate doses of inhaled air pollution.

### Data analysis

We used Wilcoxon signed-rank tests to test differences between the simultaneously measured air pollution concentrations in the various transport modes and the urban background concentration, because parametric *t*-tests were influenced by outliers. We defined level of significance as *p* < 0.05. For the continuous PNC and PM_2.5_ data, we compared 2-hr mean values. For comparisons between measurements in transport and at the urban background location and between two modes that were measured simultaneously, we calculated ratios for each sampling day and calculated median and interquartile ranges of the daily ratios.

To compare measurements that were taken on different sampling days, we corrected air pollution concentrations for absolute differences between the mean background concentrations during all sampling days and the background concentration of each sampling day, following previous studies (Cyrys et al. 2003). We used Kruskal–Wallis tests to test for differences between transport modes. We only used days with complete data sets in the analyses, and all analyses were performed using SAS (version 9.1; SAS Institute Inc., Cary, NC, USA).

## Results

### Short-term exposure variability

**Figure 2:**
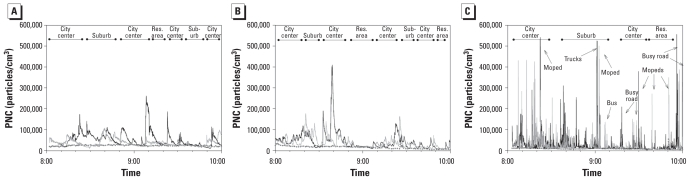
PNCs. (*A*) Bus sampling day, 22 January 2008. Black, diesel bus; gray, electric bus; dotted line, urban background. The diesel bus left and arrived about 15 min later than the electric bus. To facilitate geographical comparison, the time of the diesel bus is shifted 15 min earlier. (*B*) Car sampling day, 18 March 2008. Black, diesel car; gray, gasoline-fueled car; dotted line, urban background. (*C*) Bicycle sampling day, 10 June 2008. Black, high-traffic route; gray, low-traffic route; dotted line, urban background. Res., residential.

Figure 2 shows examples of the observed temporal variations of the PNCs found during commuting by car, bus, and bicycle. The PNCs on both bicycle routes were characterized by short, high peaks of 400,000–500,000 particles/cm^3^. Using the voice recorder data, we found that some of the peaks during cycling could be attributed to passing mopeds and buses and to cycling on busy roads. PNCs in cars and buses showed fewer, lower peaks, mostly < 200,000 particles/cm^3^. Peaks in cars and buses typically lasted up to 5 min.

PNCs were clearly higher in the city center where streets are enclosed by high buildings on both sides, which causes a street canyon effect [see Supplemental Material, Figure 3 (doi:10.1289/ehp.0901622)].

**Figure 3:**
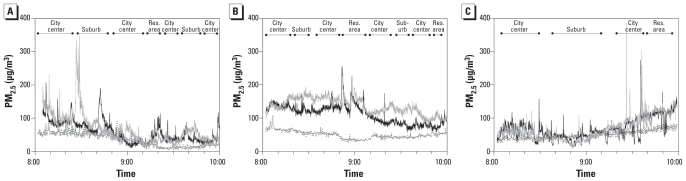
PM_2.5_. (*A*) Bus sampling day, 15 April 2008. Black, diesel bus; gray, electric bus; dotted line, urban background. The diesel bus left and arrived about 15 min later than the electric bus. To facilitate geographical comparison, the time of the diesel bus is shifted 15 min earlier. (*B*) Car sampling day, 5 June 2008. Black, diesel car; gray, gasoline-fueled car; dotted line, urban background. (*C*) Bicycle sampling day, 29 May 2008. Black, high-traffic route; gray, low-traffic route; dotted line, urban background. Res., residential.

Illustrations of real-time PM_2.5_ concentrations show short peaks of concentrations exceeding 200 μg/m^3^ (Figure 3). Mean PM_2.5_ concentrations show less clear patterns of higher concentrations in the city center than did the PNCs [see Supplemental Material, Figure 4 (doi:10.1289/ehp.0901622)].

### Differences in exposure between modes of transport and background

**Figure 4:**
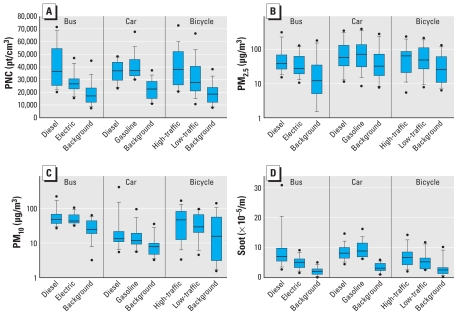
Exposures in modes of transport and at the urban background location on corresponding sampling days. (*A*) Two-hour average PNCs (particles/cm^3^). Two-hour average PM_2.5_ (*B*), PM_10_ (*C*), and soot (*D*; fraction of PM_10_). Box-and-whisker plots indicate lower and upper quartiles (box), median (line), 10–90th percentiles (whiskers), and minimum and maximum values (circles).

Figure 4 and Table 1 summarize exposure levels during commuting. Levels of all measured pollutants were significantly higher for all modes of transport compared with urban background levels. We found the smallest contrast between commuting and background concentrations for PM_10_ and the largest contrast for soot. Absolute PM_2.5_ concentration levels should be interpreted with care, because concentrations from the light-scattering method were frequently higher than the gravimetrically determined PM_10_ concentrations. Differences between in-traffic exposure and background PNC exposures are smaller for 2-hr median values than for 2-hr mean values, especially for bicycles. This is explained by the many peaks occurring during bicycle rides that affected mean values more than median values. For PM_2.5_, differences between median and mean values are small, reflecting fewer peaks in PM_2.5_ [see Supplemental Material, Table 1 (doi:10.1289/ehp.0901622)].

Soot concentrations and PNCs were higher in diesel buses than in electric buses (Table 1, Figure 4). On 7 of the 15 bus sampling days, the diesel buses were retrofitted with diesel particulate filters. Differences in pollutant concentrations measured on the diesel buses with and without filter were small and not significant. For example, the median ratio for soot concentrations in diesel buses compared with electric buses was 1.3, both with and without filters on the diesel buses.

Concentrations of all pollutants in gasoline-fueled cars were similar to concentrations in diesel cars. Sixteen different cars of six models were used throughout the study, so comparisons of different car models were not possible. The average soot concentration ratio of diesel to gasoline-fueled cars was 0.87 (range, 0.65–1.19) for the 13 days with diesel cars equipped with particulate filters, and 1.00 and 1.16 on the 2 days with diesel cars without filters.

Soot levels and PNCs were higher along high-traffic bicycle routes than along low-traffic bicycle routes.

Average meteorological conditions differed between the three types of sampling days [see Supplemental Material, Table 2 (doi:10.1289/ehp.0901622)]. There was more rainfall and higher wind speed on bus sampling days. Background concentrations of PNCs, PM_2.5_, and soot were significantly lower on bus sampling days than on car and bicycle sampling days. Background PM_10_ was significantly lower on car sampling days compared with bus and bicycle sampling days.

After correction for differences in background levels, PNC was highest along the high-traffic bicycle route and lowest in electric buses (Table 2). PM_10_ exposure was highest in diesel cars and diesel buses and lowest along both bicycle routes. Soot exposure was highest in diesel buses and gasoline-fueled cars and lowest along both bicycle routes and in electric buses. PM_2.5_ showed a different pattern: concentrations along the bicycle routes and in cars were higher than in buses. PM_2.5_ results were sensitive to RH, probably related to the applied correction factor. When only taking into account samples with mean RH < 90%, PM_2.5_ differences between diesel and electric buses and between diesel and gasoline-fueled cars remained significant, but differences among buses, cars, and bicycles disappeared.

Relative differences in PNC and PM_2.5_ exposures between diesel and electric buses, diesel, and gasoline-fueled cars and the two bicycle routes were similar in the first and second (less busy) hour of the commute. Buses and cars drove the same route, but in the second hour the buses drove more slowly and thus less of the route than did the cars. The differences in PNC and PM_2.5_ exposures between buses and cars were the same in the first hour compared with the full 2 hr.

### Differences in inhaled doses

Because of their increased physical activity, minute ventilation of cyclists was higher than that of car and bus passengers. In this study the estimated minute ventilation of cyclists was on average 23.5 L/min, varying from 11.6 to 47.7 L/min between the 34 men and women. The average minute ventilation of car passengers was 11.8 L/min, varying from 5.1 to 20.9 L/min, and the average minute ventilation of bus passengers was 12.7 L/min, varying from 5.4 to 19.5 L/min (Zuurbier et al. 2009).

On average, the minute ventilation of cyclists was 2.1 times higher than that of car passengers and 2.0 times higher than that of bus passengers. Minute ventilation of bus passengers was 1.1 times higher than that of car passengers.

Inhaled doses of all air pollutants were highest in cyclists (Table 2). Inhaled doses of PNC and PM_2.5_ of cyclists was twice as high as that of car and bus passengers, whereas inhaled doses of PM_10_ and EC (calculated using the soot levels) were substantially less than twice as high. PM_10_ dose was higher for bus than car passengers, whereas EC doses were comparable for passengers of diesel buses and both cars and lowest for passengers of electric buses.

## Discussion

Exposures to PNC, soot, PM_10_, and PM_2.5_ during commuting were significantly elevated compared with urban background concentrations. Exposures were higher in diesel buses than in electric buses and higher along high-traffic bicycle routes compared with low-traffic bicycle routes, especially for soot and PNC. Exposure levels in diesel and gasoline-fueled cars were comparable. PM_10_ and soot exposures of cyclists were lowest, whereas PNC exposure in electric buses was lowest. Because of their increased minute ventilation, the inhaled doses of all studied air pollutants were highest for cyclists. Inhaled doses of electric bus passengers were lowest for all studied air pollutants except for PM_10_.

### Comparison with urban background

The substantial contrast in commuters’ exposure compared with the urban background is consistent with previous studies (Kaur et al. 2007). Our study has added a comparison made simultaneously with the same equipment, thus ruling out potential bias related to temporal variation and sampling method.

The PM_2.5_ contrast is larger than found in previous studies. This could be related to the measurements being performed in the rush hour only versus typically daily samples in the fixed-site monitoring studies or to sampling errors of the DataRAMs. We corrected PM_2.5_ readings for RH. When omitting PM_2.5_ values with RH > 90%, differences among buses, cars, and bicycles disappeared.

### Differences between diesel and electric buses, diesel and gasoline-fueled cars, and bicycle routes

PNC and soot levels were 32% and 46% higher in diesel buses compared with electric powered buses. Diesel buses have not been compared with electric buses before. In-bus exposures are affected by the bus doors opening frequently to let passengers on and off the bus, letting in the air pollution from outside. For diesel buses, a portion of the diesel exhaust of the bus itself contributes to the in-bus exposure. The contrast agrees with the self-pollution of up to 30% reported for U.S. school buses (Adar et al. 2008; Behrentz et al. 2004). The smaller difference in PM_10_ exposure between diesel and electric buses can be explained by passengers causing resuspension of PM_10_ in both types of buses (Song et al. 2009). Differences between buses with and without retrofitted particulate filters were small and not significant, but the number of measurements may not have been sufficient to detect differences.

Our study suggests that the use of clean buses may be beneficial both for urban air quality and for bus passengers.

We found no significant differences between levels of air pollutants in diesel and gasoline-fueled cars, except for soot: In-car exposures to soot were lower in diesel cars than in gasoline-fueled cars. Possibly because of the limited number of samples in diesel cars without particulate filters, we did not detect a potential impact of particulate filters on the soot exposure in diesel cars. The major difference between cars and buses is that doors do not open regularly, so infiltration of exhaust from the car itself is less likely. We used new cars (maximum of 6 months of use). In older diesel cars, exposure levels might be higher, but from this study we conclude that for new cars there is no difference in PM exposure inside the vehicles. The rental cars were all about the same size, but not of the same model. Thus, measured air pollution levels are representative of these types of cars in general.

Cyclists on the high-traffic route were exposed to 40% higher levels of PNC and 35% higher levels of soot compared with cyclists on the low-traffic route. Exposure to PM_10_ and PM_2.5_ did not differ significantly. Kaur et al. (2005) found a 40% difference in PNC exposure among cyclists and pedestrians who traveled along high- versus low-traffic roads in Central London. Strak et al. (2009) found differences in PNC and soot of 60% and 40%, respectively, between two cycling routes with different traffic intensities, but no difference in PM_2.5_. Adams et al. (2001b) found differences in PM_2.5_ of 20–50% between low- and high-traffic bicycle routes in Central London.

Contrasts in commuters’ exposures in this study were slightly smaller than in most other studies, probably because the exposure levels on the high-traffic route were lower, especially lower than in Central London. Despite efforts to select streets with very low traffic, sections of the low-traffic bicycle route were used as a shortcut to avoid the congested major streets during morning rush hour. The car-free roads in the route were frequently used by mopeds, which have been shown to be an important source of cyclists’ exposure to PNCs (Boogaard et al. 2009).

By choosing a low-traffic route, cyclists can reduce their air pollution exposure. However, within the current Dutch infrastructure it is not easy to avoid all contact with motorized traffic.

### Differences in exposure between modes of transport

Compared with cyclists, PM_10_ exposures of bus and car passengers were 60% and 20% higher, respectively. The small study of Rank et al. (2001) detected 70% more total PM exposure in cars compared with exposure on bicycles, similar to our data. Resuspension of coarse PM probably partly explains the difference in exposure. The significantly higher soot concentration in cars and diesel buses versus cyclists is in agreement with results of Adams et al. (2002), who measured 70% higher EC exposures in cars compared with cyclists and 20% higher EC in buses compared with cyclists.

Exposures to PM_2.5_ were comparable in all modes in our study. In electric buses levels were slightly lower and in gasoline-fueled cars slightly higher than in all other modes. Kaur et al. (2005) and Boogaard et al. (2009) also found only small differences in PM_2.5_ concentrations between cars, diesel buses, and bicycles. Average differences in PNC exposure were small among cyclists, car, and diesel bus passengers in the present study. Diesel bus passengers and cyclists along the high-traffic route experienced 10–30% higher PNC exposure than did car passengers and cyclists on the low-traffic route. PNC exposure in electric buses was clearly lowest. Boogaard et al. (2009) reported slightly higher PNC exposure in cars compared with cyclists. Kaur et al. (2005) reported slightly higher PNC exposure of bus and car passengers compared with cyclists. Absolute PNC levels were much higher in London, as can be expected because of higher traffic intensity in London compared with the relatively small city of Arnhem. PNCs on bicycle routes were characterized by short, high peaks, whereas PNCs in cars and buses showed fewer, lower peaks. Indicators of frequency distribution, such as the 95th percentile of the values, may reflect exposure differences more completely.

### Inhaled doses of air pollutants

Inhaled pollution doses were highest for cyclists, because of the higher minute ventilation (Zuurbier et al. 2009). We found almost two times higher pollution doses in cyclists for PNC; the differences in soot (EC) and PM_10_ were more modest. In view of the heavy equipment slowing down the speed of the cargo bike, the speed of cycling in the study was relatively low (12 km/hr) compared with average cycling speeds, which are around 15 km/hr, so this study may underestimate minute ventilation levels and consequently the inhaled dose of air pollutants during normal cycling. However, in the one previous study that compared minute ventilation of cyclists (cycling at 17 km/hr) with that of car drivers (van Wijnen et al. 1995), the ratio was 2.3, comparable to our average ratio of 2.1.

Traveling time also influences the doses of inhaled air pollutants. Because traveling time depends on the route and on the time of day—during rush hour cyclists and buses using separate bus lanes may travel faster than cars, whereas in most other situations cars travel faster—we did not take this into account in our study.

### Potential policy implications

This study shows that exposure to air pollutants is significantly lower in electric powered buses than in diesel buses. The use of clean buses, such as electric buses, is therefore beneficial not only for outdoor air quality but also for bus passengers. Policy makers are encouraged to increase the use of clean buses, such as electric buses.

Cyclists are exposed to air pollutants from surrounding traffic. The higher minute ventilation of cyclists especially increases the inhaled doses of air pollutants. Health implications of exposure to short, high peaks during cycling instead of the lower, longer peaks in cars and buses are not clear but could be important (Peters et al. 2004). Because the positive health effects of cycling (Baumann 2004; de Geus et al. 2009, 2008; Hendriksen et al. 2000) likely outweigh the health risks of increased pollution loads, and because cyclists do not emit any air pollutants and thus contribute to clean air, cycling should not be discouraged. Cyclists should be encouraged to choose for low-traffic routes by making them aware of the potential health benefits and by route planners with options to choose for low-traffic routes. City planners should create bicycle lanes with less (preferably no) contact with motorized traffic. In view of the intimate mixing of bicycles and mopeds in the Netherlands, further improvements can be expected from the replacement of spark engine by electric mopeds.

## Figures and Tables

**Table 1:** PNCs, PM_2.5_, PM_10_, and soot concentrations during commuting by bus, car, or bicycle.

Pollutant/mode of transport	*n* (days)	Mean ± SD	Median	Mode:background ratio^a^	Mode 1:mode 2 ratio^b^
PNC (particles/cm^3^)^c^					
Diesel bus	13	43,235 ± 17,388	38,536^d^,^e^	2.54 (1.16–3.33)	1.22 (1.07–2.01)
Electric bus	13	28,602 ± 8,399	29,245^e^	1.63 (1.12–2.35)	—
Urban background	13	18,908 ± 10,697	13,913	—	—

Diesel car	14	37,129 ± 8,262	37,831^e^	1.62 (1.50–2.08)	0.96 (0.85–1.08)
Gasoline car	14	40,526 ± 10,142	37,451^e^	1.71 (1.45–2.44)	
Urban background	14	22,275 ± 7,935	22,756	—	—

High-traffic bicycle	15	48,939 ± 19,039	46,570^e^,^f^	2.25 (1.63–2.49)	1.33 (1.09–1.40)
Low-traffic bicycle	15	39,576 ± 18,178	33,159^e^	1.77 (1.22–2.17)	—
Urban background	15	23,798 ± 10,523	23,360	—	—

PM_2.5_ (μg/m^3^)^c^					
Diesel bus	10	68.7 ± 91.7	39.1^d^,^e^	2.98 (1.64–8.91)	1.41 (1.04–1.73)
Electric bus	10	40.5 ± 32.1	27.7^e^	2.67 (1.23–5.60)	—
Urban background	10	33.6 ± 55.8	9.6	—	—

Diesel car	14	101.3 ± 103.9	59.7^e^,^g^	1.57 (1.37–2.22)	0.88 (0.64–1.08)
Gasoline car	14	114.8 ± 118.1	73.6^e^	1.95 (1.44–2.69)	—
Urban background	14	67.5 ± 84.1	32.6	—	—

High-traffic bicycle	16	72.3 ± 67.0	49.8^e^	2.11 (1.64–3.20)	0.98 (0.81–1.29)
Low-traffic bicycle	16	71.7 ± 65.5	65.2^e^	2.26 (1.51–3.50)	—
Urban background	16	37.8 ± 41.0	22.5	—	—

PM_10_ (μg/m^3^)					
Diesel bus	11	68.5 ± 53.6	46.7^e^	2.49 (1.37–3.70)	0.99 (0.88–1.23)
Electric bus	11	53.2 ± 23.2	43.5^e^	2.24 (1.11–3.70)	—
Urban background	11	28.2 ± 17.0	24.6	—	—

Diesel car	14	78.5 ± 101.5	49.8^e^	1.44 (1.09–1.82)	1.06 (0.87–1.18)
Gasoline car	14	58.7 ± 34.6	47.9^e^	1.26 (1.12–1.68)	—
Urban background	14	39.5 ± 21.1	34.7	—	—

High-traffic bicycle	15	38.8 ± 14.1	39.3^e^	1.10 (1.04–1.58)	1.07 (0.87–1.18)
Low-traffic bicycle	15	37.2 ± 11.6	37.0^e^	1.13 (0.99–2.03)	—
Urban background	15	30.9 ± 16.3	29.2	—	—

Soot (× 10^−5^/m)					
Diesel bus	12	9.0 ± 7.2	7.4^d^,^e^	3.66 (2.79–9.40)	1.34 (1.25–2.12)
Electric bus	12	5.1 ± 2.1	5.1^e^	2.87 (2.23–3.22)	—
Urban background	12	2.0 ± 1.3	1.8	—	—

Diesel car	15	8.2 ± 2.7	7.9^e^,^g^	2.88 (2.02–3.76)	0.95 (0.75–1.00)
Gasoline car	15	9.3 ± 3.0	9.0^e^	2.91 (2.35–4.74)	—
Urban background	15	3.1 ± 1.5	2.7	—	—

High-traffic bicycle	16	6.6 ± 3.2	6.6^e^,^f^	2.42 (2.02–3.26)	1.25 (1.17–1.45)
Low-traffic bicycle	16	5.3 ± 2.8	4.9^e^	2.00 (1.72–2.28)	—
Urban background	16	3.1 ± 2.7	2.4	—	—

Wilcoxon signed rank tests were used to test differences between the simultaneously measured transport modes and background.

a Median and interquartile range (in parentheses) of daily ratio of mode to background.

b Median and interquartile range (in parentheses) of daily ratio of mode 1 (mode in first line, e.g., diesel bus) to mode 2 (mode in second line, e.g., electric bus).

c Median values of 2-hr mean. Minimum and maximum values are the lowest and highest 2-hr mean occurring during the sampling days.

d *p* < 0.05 compared with electric bus.

e *p* < 0.05 compared with urban background.

f *p* < 0.05 compared with low-traffic bicycle route.

g *p* < 0.05 compared with gasoline car.

**Table 2:** Exposure levels of PNC, PM_2.5_, PM_10_, and soot.

Pollutant/mode of transport	*n* (days)	Median exposure^a^	Median dose^b^	Mode:low-traffic bicycle ratio
PNC		Particles/cm^3^^c^	Particles/hr^c^ (×10^9^)	

Diesel bus	13	44,985^d^	34.3	0.68
Electric bus	13	31,833^e^,^f^,^g^	24.3	0.48
Diesel car	14	35,351^g^	25.0	0.50
Gasoline car	14	38,844^d^	27.5	0.55
High-traffic bicycle	15	42,088^h^,^i^	59.3	1.18
Low-traffic bicycle	15	35,815^h^	50.5	—

PM_2.5_		μg/m^3^^c^	μg/hr^c^	

Diesel bus	12	73.0	55.7	0.60
Electric bus	12	59.8^f^	45.6	0.49
Diesel car	14	73.4	51.9	0.56
Gasoline car	14	87.9^d^	62.3	0.67
High-traffic bicycle	16	71.2	100.4	1.08
Low-traffic bicycle	16	66.1	93.3	—

PM_10_		μg/m^3^	μg/hr	

Diesel bus	11	60.5^g^,^i^	46.1	0.88
Electric bus	11	56.7^i^	43.2	0.82
Diesel car	14	45.2	32.0	0.61
Gasoline car	14	42.4	30.0	0.57
High-traffic bicycle	15	35.6^e^	50.2	0.96
Low-traffic bicycle	15	37.2^d^,^e^	52.5	—

Soot		(× 10^−5^/m)	EC (μg/hr)	

Diesel bus	12	7.7^d^,^i^	9.2	0.88
Electric bus	12	5.7^i^,^f^,^h^	6.8	0.65
Diesel car	15	7.7^d^,^g^,^i^	8.6	0.82
Gasoline car	15	8.2^d^,^g^,^i^	9.1	0.87
High-traffic bicycle	16	6.2^f^,^h^,^i^	13.6	1.30
Low-traffic bicycle	16	4.8^d^,^e^,^f^,^g^,^h^	10.5	—

Wilcoxon signed-rank tests were performed to test differences between simultaneously measured transport modes. Kruskal–Wallis tests were performed to test differences between nonsimultaneously measured modes.

a Concentrations corrected for differences in background concentrations.

b Concentrations corrected for differences in background concentrations and using the average minute ventilation of 23.5 L/min for cyclists, 11.8 L/min for car passengers, and 12.7 L/min for bus passengers.

c Median values of 2-hr mean.

d *p* < 0.05 compared with electric bus.

e *p* < 0.05 compared with diesel bus.

f *p* < 0.05 compared with gasoline-fueled car.

g *p* < 0.05 compared with high-traffic bicycle route.

h *p* < 0.05 compared with diesel car.

i *p* < 0.05 compared with low-traffic bicycle route.
